# O024. Transcutaneous supraorbital nerve stimulation enhances somatosensory thalamic activity in migraine between attacks: a central mechanism of clinical efficacy?

**DOI:** 10.1186/1129-2377-16-S1-A160

**Published:** 2015-09-28

**Authors:** Davide Di Lenola, Gianluca Coppola, Mariano Serrao, Cherubino Di Lorenzo, Francesco Pierelli

**Affiliations:** 1 Department of Medico-Surgical Sciences and Biotechnologies, “Sapienza” University of Rome Polo Pontino, Latina, Italy; 2 Department of Neurophysiology of Vision and Neuro-ophthalmology, G.B. Bietti Foundation IRCCS, Rome, Italy; 3 Don Carlo Gnocchi Onlus Foundation, Milan, Italy

## Background

In a recent randomized double-blind sham-controlled study the Cefaly®, a novel transcutaneous supraorbital electrostimulation device, has been successfully used as a prophylactic treatment for episodic migraine. The possible mechanisms of action through which the device is able to induce clinical improvement in migraine are not known. In the present study, we investigated whether Cefaly® may act centrally at the thalamocortical/cortical level.

## Methods

To explore the central mechanisms of action of Cefaly®, we recorded the somatosensory evoked potentials (SSEPs) before, and in the subsequent two times after one session of supraorbital stimulation lasting 20 min in 10 migraine without aura patients between attacks. We measured the N20-P25 amplitudes on the low-frequency-SSEP, and, after applying a band-pass filter (450-750 Hz), maximal peak-to-peak amplitudes of the pre-synaptic, reflecting thalamocortical activity, and post-synaptic, reflecting primary cortical activation, high-frequency oscillations (HFOs).

## Results

Pre-synaptic HFO amplitudes, reflecting somatosensory thalamocortical activity, significantly increased after the stimulation (from 0.035 V to 0.058 V, p < 0.01), whereas both the low-frequency N20 SSEP component and post-synaptic HFOs were unaffected.

## Conclusions

Present data might support the hypothesis that Cefaly® acts centrally through increased thalamocortical activity induced by the neurostimulation. It is of obvious interest to verify whether these device-induced changes might persist in the long-term after 3-month daily preventive stimulation, and if they follow clinical improvement.

Written informed consent to publish was obtained from the patient(s).

